# Hydroxychloroquine and Fabry Disease: Three Case Reports Examining an Unexpected Pathologic Link and a Review of the Literature

**DOI:** 10.1155/2022/2930103

**Published:** 2022-07-11

**Authors:** Rouhin Sen, Kathleen Borghoff, Kirk W. Foster, Stanley J. Radio, Alan Erickson, Michelene Hearth-Holmes

**Affiliations:** ^1^University of Colorado Denver, Division of Rheumatology, Barbara Davis Center, Mail Stop B115, 1775 Aurora Court, Aurora 80045, Colorado, USA; ^2^University of Nebraska Medical Center, Division of Diabetes, Endocrinology and Metabolism, 984130 Nebraska Medical Center, Omaha 68198, NE, USA; ^3^University of Nebraska Medical Center, Department of Pathology and Microbiology, 985900 Nebraska Medical Center, Omaha 68198, NE, USA; ^4^University of Nebraska Medical Center, Division of Rheumatology, 986270 Nebraska Medical Center, Omaha 68198, NE, USA

## Abstract

**Background:**

Hydroxychloroquine is an effective and widely used treatment in multiple autoimmune connective tissue diseases that gained a lot of publicity in the coronavirus disease 2019 (COVID-19) pandemic. Our case reports are unique in that they explore the rare and sometimes overlooked effects of this drug on multiple organ systems, specifically the kidney, cardiac muscle, and skeletal muscle. We include key histologic features in images which aid in identifying and distinguishing hydroxychloroquine toxicity from mimickers. Lastly, we report the very interesting similarity in the intracellular action of hydroxychloroquine to the pathology of Fabry disease (and its associated lysosomal enzyme, *α*-galactosidase A). *Case Presentation*. We will examine the case presentations of three female Caucasian patients: a 22-year-old with lupus nephritis class V, a 72-year-old with long-standing systemic lupus erythematosus, and a 74-year-old with undifferentiated connective tissue disease. All three patients were on hydroxychloroquine therapy for varying amounts of time with histologic evidence of hydroxychloroquine toxicity that is three is present in histological samples of the kidney, the heart, and the skeletal muscle.

**Conclusions:**

Hydroxychloroquine is a very important and beneficial medication used for various autoimmune connective tissue diseases. Clinicians should be aware of the rare but sometimes serious side effects that can result from the medication, which at times can mimic manifestations of the connective tissue disease itself or Fabry disease. A thorough investigation should be performed in these cases to properly elucidate the cause followed by the appropriate targeted therapy.

## 1. Introduction

Hydroxychloroquine (HCQ) was thrust into the public spotlight due to the pandemic caused by the severe acute respiratory syndrome coronavirus 2 (SARS-CoV-2). Though its efficacy against SARS-CoV-2 is questionable, the drug certainly has gained tremendous popularity leading to subsequent demand and worldwide shortages.

HCQ is an old drug that is quite well-known to rheumatologists. The drug traces its ancestry to quinine, a compound present in the bark of the cinchona, “the fever tree.” Quinine was first isolated in the 1800s, but cinchona bark extracts were in use for at least two centuries prior in Peru and Europe for the treatment of “ague” (malaria). Quinine supplies were reduced during the ravage of World War II leading to the development of synthetic substitutes—chloroquine and quinacrine [1].

Because of perceived toxicity, chloroquine and quinacrine were largely ignored for years until postwar studies in the United States showed their tremendous value in treating malaria [1]. Chloroquine emerged as one of the principal weapons in the World Health Organization's fight against the disease. Around the same time, anecdotal reports from veterans described improvement in symptoms from systemic lupus erythematosus (SLE) and rheumatoid arthritis (RA) leading up to a 1951 study in Lancet describing the benefits of quinacrine on several patients with lupus [2].

Within the next few years, quinacrine, chloroquine, and the newer HCQ (differing from chloroquine by one hydroxyl group) were used to treat SLE and RA [1]. For various reasons including side effects and efficacy, chloroquine and quinacrine fell out of favor and were not widely prescribed. HCQ, an oral cost-effective medication with excellent bioavailability, became a cornerstone drug and ubiquitous in the treatment of several connective tissue diseases and eventually approved by the US Food and Drug Administration for discoid lupus, SLE, and RA [3]. For many of these conditions including SLE, HCQ reduced clinical flare while improving survival, undoubtedly earning its place on the World Health Organization's “List of Essential Medicines” as a disease-modifying agent used in rheumatic disorders.

In its more than half-century of use, HCQ has been generally considered safe, well-tolerated, and with minimal interactions; it notably requires regular eye exams for retinopathy screening and commonly causes nausea and mild diarrhea. Only isolated cases of nephropathy, cardiomyopathy, and skeletal myopathy have been reported. This case series examines some of these lesser-known but serious side effects of HCQ, related histology and pathology, and most interestingly, similarity to the lysosomal storage disorder, Fabry disease.

## 2. Case Reports

### 2.1. Case One

A 22-year-old Caucasian female 10 weeks pregnant was admitted with complaints of nausea, vomiting, diarrhea, and fevers. She has a known history of systemic lupus erythematosus and Sjogren syndrome diagnosed at the age of 17; treatment has been primarily with HCQ since the time of diagnosis. At the time of admission, she was on HCQ 400 mg daily. Previously, she had tried azathioprine which was not tolerated due to vomiting and mycophenolate mofetil which was stopped prior to conception. In the past, she has also required treatment with both high- and low-dose oral and intravenous steroids for flares and inflammatory joint pains. She was on prednisone 15 mg daily at the time of admission.

Admission serology was as follows: anti-nuclear-antibody (ANA) positive (1 : 640, homogenous pattern), anti-SSA 201 AU/mL, anti-SSB 664 AU/mL, anti-Smith 411 AU/mL, anti-RNP 306 AU/mL, anti-dsDNA 290 AU/mL, and anti-histones 276 AU/mL. Complete blood count showed stable normocytic normochromic anemia and complete metabolic panel was significant only for a potassium level of 3 mg/dL which was replaced. Complement levels were within normal limits. Her urine analysis showed increased proteins at greater than 300 mg/dL. Subsequent urine protein to creatinine ratio was 5.1 which was a significant change from outpatient labs obtained approximately 3 months prior to admission.

**Figure 1:**
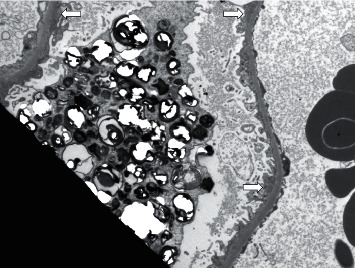
Electron micrograph showing subepithelial electron-dense deposits consistent with class 5 lupus nephritis and intracytoplasmic lamellated bodies (also known as curvilinear bodies-white arrows) within the overlying podocyte. Original magnification 1000×.

During her hospital course, her symptoms showed improvement with symptomatic treatment and fluids. Due to the increased proteinuria and to assess renal involvement of lupus, she agreed to undergo a kidney biopsy. Pathology showed Class V membranous lupus nephritis. In addition to these findings, electron microscopy showed that intracellular inclusions were found within many podocytes and some tubular epithelial cells (Figure 1). The inclusions were composed of lamellated electron-dense material with the appearance of myelin bodies. The findings were consistent with what had been described in patients receiving HCQ therapy. HCQ was stopped and she was put on increased doses of oral prednisone; her proteinuria resolved two months later. She was restarted on mycophenolate mofetil alone after her pregnancy came to term.

### 2.2. Case Two

A 72-year-old Caucasian female was transferred from an outside hospital for progressively worsening heart failure (New York Health Association Class IV) and evaluation of cardiac replacement therapy and ventricular assistive device. Her past medical history was significant for SLE diagnosed 50 years prior to our initial encounter. Her disease was controlled well with HCQ 200 mg daily for more than 20 years. For flares, she reported taking low-dose oral prednisone as needed.

Her initial admission vitals were stable. Physical exam showed a cachectic appearing elderly female with ulnar drift across her metacarpal joints which were completely reducible. The exam also revealed lower extremity bunions bilaterally with cock-up deformities. No joint effusions were noted. She had 2+ pedal edema bilaterally and hyper- and hypopigmentation on her lower extremities.

Her initial labs were significant for pancytopenia which from outside records seemed chronic. A complete metabolic panel was significant for an elevated creatinine of 1.9 mg/dL but otherwise, normal electrolytes.

Transthoracic echocardiogram reported dilated left ventricle with a globally depressed ejection fraction of 15%, grade III diastolic dysfunction, severe mitral regurgitation, severe tricuspid regurgitation, and elevated right ventricular systolic pressure (RVSP). Previous but recent cardiac catheterization findings were consistent with a nonischemic cause for her cardiomyopathy. Right heart catheterization was performed to evaluate for pulmonary artery hypertension and showed a pulmonary artery wedge pressure of 47 mmHg.

**Figure 2:**
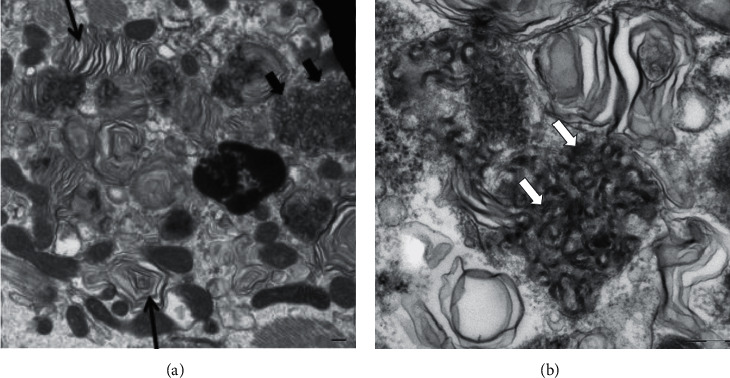
On the left (a), an electron micrograph of endomyocardial biopsy with myeloid bodies (single black arrows) is present including a “zebra” type in the upper part of the figure. Curvilinear bodies (white double arrows) are also present. On the right (b), higher magnification of curvilinear bodies.

An endomyocardial biopsy was performed to evaluate the cause of her cardiomyopathy and showed mild ventricular myocyte hypertrophy and focal mild interstitial fibrosis. Myeloid bodies and curvilinear bodies were present on ultrastructural examination (Figure 2). The finding of curvilinear bodies was known to be highly specific for HCQ toxicity, and the drug was discontinued. The patient was deemed a poor candidate for a ventricular assistive device due to poor nutritional status and thrombocytopenia. She was discharged home on milrinone infusion and life-vest but passed away shortly after discharge.

### 2.3. Case Three

A 76-year-old Caucasian female was seen in the clinic for progressive weakness in her upper and lower extremities. She was known to have a history of an undifferentiated connective tissue disorder manifested by inflammatory arthritis, Raynaud phenomenon, high ANA titer (1 : 640, homogenous pattern), dsDNA of 70 AU/mL, anti-RNP IgG >8 AU/mL, and anti-Smith antibodies 2.3 AU/mL. She also had a history of Sjogren syndrome confirmed via lip biopsy and positive Schirmer test but with negative anti-SSA/SSB.

She was taking HCQ 200 mg twice daily, methotrexate 7.5 mg weekly, and prednisone 5 mg daily for her rheumatic diseases. She was on HCQ for approximately 4 years. She was recently admitted inpatient for muscle weakness but continued to have trouble with activities of daily living following discharge despite physical therapy.

Her initial vital signs in the clinic were stable. Her physical exam revealed 4/5 strength in bilateral deltoids, biceps, triceps, and iliopsoas and 5/5 strength elsewhere. Her creatine kinase was normal at 109 U/L, and aldolase was slightly elevated at 8.6 U/L (the normal range is 1.5–8.1 U/L) in a hemolyzed sample. Repeat aldolase was normal at 7.5 U/L.

**Figure 3:**
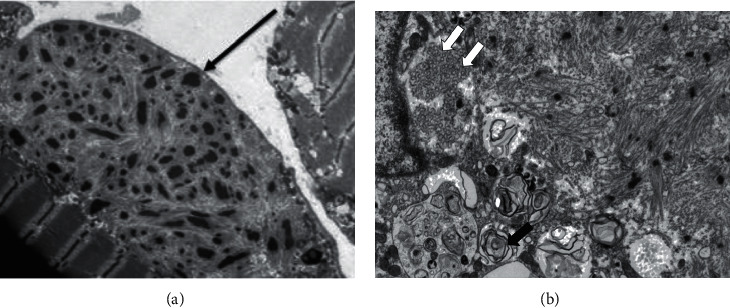
On the left (a), an electron micrograph of skeletal muscle biopsy with Nemaline rod (black arrow). On the right (b), an electron micrograph of the myeloid body, black arrow and curvilinear bodies (white double arrows).

She had electromyography (EMG) which showed chronic, primarily axonal, sensory motor peripheral neuropathy of an estimated moderate degree. Of note, the EMG was limited in quality due to a recent history of cellulitis in her lower extremities. A muscle biopsy (Figure 3) showed more than 50% lobulated myofibers, some with excessive mitochondrial activity, small numbers of myofibers with nemaline rods, lysosomal curvilinear bodies, and fiber type grouping, all consistent with chronic denervation and reinnervation. The lysosomal curvilinear bodies were known to be consistent with HCQ therapy. Her HCQ was stopped, and she began following up with neurology for nemaline myopathy.

## 3. Discussion

The immunomodulatory properties of HCQ are likely due to its effects on the innate immune system and acidic cellular compartments [4]. HCQ is a weak amphiphilic base that can easily cross the plasma membrane into acidic cytoplasmic vesicles, such as lysosomes, where it is protonated and trapped. The accumulation of HCQ raises the pH in vesicles interfering with functions that are dependent upon acidic environments. This interferes with the action of Toll-like receptors and their ligands which plays an important role in receptor recycling, intracellular processing, and the secretion of proteins [3]. This interference leads to decreased production of cytokines and other inflammatory mediators, decreased lymphocytic proliferation and natural killer cell activity, altered antibody production, and interference with antigen processing [5]. Thus, through various actions, HCQ decreases the activity of the innate immune system in autoimmune diseases [3].

### 3.1. Relation of HCQ to Fabry Disease

The increased intravacuolar pH inhibits the action of lysosomal enzymes, one of which is *α*-galactosidase A [6]. This very same enzyme is deficient in the rare X-linked recessive lysosomal disorder, Fabry disease. The absence of the enzyme in Fabry disease causes an accumulation of intracellular glycolipid globotriaosylceramide in cells of the kidney, vascular endothelium, cardiac, skeletal, and smooth muscles [7]. Accumulation of this globotriaosylceramide causes impairment in the function of these tissues. The rare side effects seen in our cases could result from a similar pathology as Fabry disease due to inhibition of *α*-galactosidase A and subsequent aggregation of globotriaosylceramide.

Renal manifestations of Fabry disease include proteinuria and unexplained renal insufficiency similar to case one. Our patient had no family history of lysosomal storage disorders making it an unlikely cause of the renal pathology. Ultrastructural kidney biopsy findings in Fabry disease include lamellated myeloid bodies and zebra bodies found in both podocytes and endothelial cells. This was once thought to be exclusive to the disease but now, has also been described in other conditions that cause renal phospholipidosis including the use of drugs such as HCQ [8]. Ultrastructural analyses have also shown a feature unique to HCQ use and never reported in Fabry disease-curvilinear inclusion bodies [9].

### 3.2. Curvilinear Inclusion Bodies

Curvilinear inclusion bodies were first described by Muller-Hocker et al. in 2003 [10]. Inclusion bodies are nuclear or cytoplasmic aggregates of proteins. A curvilinear inclusion body is this protein aggregate in a lamellated and twisted structure surrounded by a membrane. Cases by Ferluga [11] and Costa et al. [8] reported that patients on HCQ therapy had histopathological and ultrastructural findings similar to Fabry disease. In these case series, the majority of patients had curvilinear inclusion bodies. Though not considered a uniform finding, this is specific to HCQ toxicity not only in the kidney but also in the cardiac and skeletal muscles.

HCQ-induced damage to cardiac and skeletal muscle is rare and hard to quantify. One study identified nearly 10% prevalence and 1.2% annual incidence of just skeletal myopathy with HCQ use [12]. Another case series reported 1 case of skeletal myopathy in 100 patient-years of therapy [13]. Risk factors for developing cardiac or skeletal myopathy include female gender, Caucasian race, older age, and renal dysfunction. Long-standing treatment with HCQ is also thought to increase the risk of myopathies with effects usually seen after 10 years of use [14]. Discontinuation of therapy is thought to improve skeletal myopathy but this process can take months given the long half-life of HCQ [3].

Cardiac and skeletal myopathy related to HCQ use and Fabry disease clinically present similarly, which is not surprising given their pathologic similarity. HCQ cardiomyopathy can manifest as systolic or diastolic heart failure, conduction anomalies, left or right ventricular hypertrophy, valvular disease, coronary artery disease, or hypertension [14]. The most common presentation for HCQ cardiomyopathy is typically heart failure. A recent case series by Tselios et al. demonstrated 8 out of 8 patients with abnormal cardiac biomarkers and left ventricular hypertrophy in patients with HCQ cardiomyopathy [15]. The question as to why HCQ causes cardiac issues may be explained by its affinity to melanocytes which have been shown to exist in the human heart [16].

HCQ skeletal myopathy and Fabry disease typically present with proximal lower extremity weakness or atrophy [17]. Serum creatine kinase and lactate dehydrogenase can be mildly elevated, which may be overlooked in the context of rheumatic diseases. Due to the higher-than-expected prevalence of HCQ skeletal myopathy, some experts have recommended monitoring for myopathies using a physical exam, serologic tests, and electromyography [18]. Biopsy of the cardiac or skeletal muscle provides a definitive diagnosis, as is the case for HCQ nephropathy.

Typical histopathologic findings in HCQ skeletal and cardiac myopathy are similar to and consistent with what is seen in Fabry disease [18]. Vacuolar myopathy is a finding in nearly all reported cases [19]. Like HCQ nephropathy, the electron microscopic finding of curvilinear inclusion bodies is considered specific for HCQ skeletal and cardiac myopathy and present in over 90% of biopsies [17, 18].

Mortality for anti-malarial induced cardiomyopathy is high; a review of cases by Tselios et al. showed mortality of 45% within 3-4 months after diagnosis [17]; in keeping with this observation, our patient did expire within months of diagnosis. Interestingly, there have been reports suggesting possible reversibility of skeletal and cardiac myopathy with cessation of the drug [19]. In some case reports, patients had improved subjective complaints such as shortness of breath or skeletal muscle weakness. There have also been reports of improved systolic function, evidence of regression of ventricular hypertrophy, and even complete resolution of pathologic changes [17] within 6 months to 2 years of drug cessation. Earlier diagnosis of HCQ cardiomyopathy may lead to better survival. Cardiac magnetic resonance imaging (MRI) using T1 mapping has been used to detect subclinical heart damage in Fabry disease, and there is some hope it can be used for early detection in those with suspected HCQ cardiomyopathy [20]. However, there are also several case reports showing no improvement of either cardiac or skeletal myopathy despite stopping HCQ due to the reservoir of the drug in deposited tissue.

It is unclear how to predict the patients who may develop HCQ nephropathy or myopathy. The development of retinopathy, which is very closely monitored in those on HCQ therapy, has not been consistently found in patients with myopathy; only about 13% of patients with myopathy had preceding or concurrent ocular disease [14, 17]. Another study has suggested that skin hyperpigmentation could be a marker for those who are at higher risk of HCQ toxicities [21] since the skin is a reservoir for the drug but this association has also not been proven.

## 4. Conclusion

There are only a few case reports of HCQ-induced nephropathy, skeletal myopathy, and cardiac myopathy in the literature. Given the use of HCQ for the past 60–70 years, the prevalence of nephropathy or myopathy related to the drug's use seems negligible. This may be because many cases are subclinical, under-recognized, or attributed to another cause such as the connective tissue disease itself. In the future, it would be interesting to investigate a correlation between partial or reduced *α*-galactosidase A activity and the higher risk of developing HCQ toxicity and if the activity level correlated inversely to the degree of toxicity. The high mortality and potential reversibility of HCQ nephropathy and myopathy emphasize the importance of recognition and consideration of alternative therapy in these patients.

## Data Availability

The data used to support the findings of this case report are included within the article.
